# The supportive conditions of wuwei leadership: Laozi, autonomy, and employee well-being

**DOI:** 10.3389/fpsyg.2026.2008668

**Published:** 2026-10-21

**Authors:** Pinghua Liu, Xinjun Gao

**Affiliations:** 1 General Education College, Guanyi Culture Research Institute, Guangdong University of Science and Technology, Dongguan, China; 2 School of Psychology, Northwest Normal University, Lanzhou, China

**Keywords:** autonomy support, employee well-being, Laozi, leadership, psychological safety, wuwei

## Abstract

Under what conditions does reduced managerial intervention support employee autonomy, and when does it become a failure of responsibility? Drawing on a selective reconstruction of wuwei in the Laozi, this Perspective develops a conditional account of leadership in dialogue with autonomy-supportive and empowering leadership. It distinguishes ancient political thought from contemporary employment relations and treats the Laozi as a conceptual resource. The proposed contribution concerns how leaders justify intervention, return decision authority, and preserve responsibility. Four supportive conditions address distinct practical problems: role clarity defines the scope of discretion, resources enable action, accessible guidance supplies assistance, and accountability transparency makes responsibility visible. These dimensions form a provisional organizational framework, not an exhaustive list or a validated scale. To make empirical testing manageable, the initial model prioritizes guidance accessibility as a moderator of the association between lower controlling intervention and autonomy satisfaction, with work vitality as the primary outcome. Withdrawal of necessary guidance and coordination is proposed to relate to strain through role ambiguity. Task risk, employee experience, and cultural and institutional contexts guide subsequent extensions. Three propositions and a staged research strategy clarify how this perspective can complement established leadership theories without presuming a distinct wuwei construct or established causal effects.

## 1 Introduction

Reduced managerial intervention can be experienced as either trust or abandonment. When leaders issue fewer instructions, employees may gain discretion over how to complete their work; they may also be left without the authority, information, or assistance needed to exercise that discretion. The visible amount of intervention therefore cannot by itself indicate whether leadership is supportive. This ambiguity matters for employee well-being because avoidant leadership has been associated with role ambiguity, stressors, and psychological distress (Skogstad et al., 2007, 2014). Goal orientations can also qualify how employees appraise laissez-faire leadership and respond in performance terms (Zhang et al., 2023). A useful account must distinguish restraint that preserves employees’ agency from withdrawal that transfers uncertainty and risk downward.

Daoist approaches to leadership already recognize both the constructive promise and the problematic uses of non-action. Studies of wuwei have examined contextual responsiveness, managerial flow, self-protection, water-like leadership, and mindful or nurturing practice (Prince, 2005; Xing and Sims, 2012; Auzoult, 2021; Tan, 2023). Recent criticism also warns against turning Daoism into an idealized and internally uniform leadership doctrine (Lei, 2025). The unresolved issue is consequently not whether wuwei can be associated with good leadership in the abstract, but which organizational conditions allow restrained intervention to function as support rather than evasion.

This Perspective develops a conditional account through a selective reading of the Laozi and a directed dialogue with organizational research. It justifies four support conditions, specifies how restraint complements established leadership theories, and prioritizes a parsimonious empirical model. The contribution is theoretical clarification and falsifiable propositions, not a systematic review, a validated measure, or evidence that the proposed causal chain has already been established.

## 2 Wuwei as a resource for rethinking leadership

The Laozi offers resources for examining control without supplying a ready-made theory of modern employment. Its claims emerge from ancient political, ethical, and cosmological debates rather than from institutions organized around contracts, professional rights, and contemporary duties of care. Applying wuwei to workplaces must therefore be a philosophical reconstruction, not an assertion of historical equivalence. The reconstruction used here selects textual concerns with coercion, possession, and agents’ capacity to act, while acknowledging that modern commitments to employee voice, informed choice, and organizational accountability require independent justification. Chinese quotations from the Laozi and Wang Bi’s commentary follow Lou Yulie’s critical edition of Laozi Daodejing zhu (Wang, 2011); citations use printed page numbers. Characters and punctuation are normalized, and translations are our own.

Chapters 17 and 51 invite reflection on accomplishment that is not appropriated by a directing authority. Chapter 17 describes a successful order in which “功成事遂,百姓皆谓我自然” [when the work is accomplished and affairs are brought to completion, the people all say, “We are so of ourselves”] (Wang, 2011, ch. 17, p. 43, authors’ translation). Chapter 51 likewise characterizes generative activity as “生而不有,为而不恃,长而不宰” [giving life without possessing; acting without presuming upon it; nurturing without ruling over] (Wang, 2011, ch. 51, p. 141, authors’ translation). Neither passage measures autonomy in the psychological sense. Together, however, they make it possible to ask whether leaders can contribute to collective accomplishment without making their own direction the sole source of order or claiming employees’ agency as managerial property.

Chapter 64 places restraint beside assistance and attention to emerging difficulty. Its phrase “以辅万物之自然,而不敢为” [to assist the myriad things in being so of themselves, without daring to act upon them] (Wang, 2011, ch. 64, p. 171, authors’ translation) connects assistance with a refusal to impose. Yet the same chapter also advises, “为之于未有,治之于未乱” [act upon it before it has come into being; bring order to it before disorder arises] (Wang, 2011, ch. 64, p. 170, authors’ translation). The tension is instructive. Noncoercive leadership need not be indifferent to developing problems; the relevant questions concern the form, timing, and justification of intervention. Contemporary application should therefore avoid making inactivity itself the goal.

An autonomy-oriented reconstruction must also acknowledge passages that resist egalitarian readings. Chapter 3 prescribes “常使民无知无欲” [always keep the people without knowledge and without desire] (Wang, 2011, ch. 3, p. 9, authors’ translation), while chapter 65 states “非以明民,将以愚之” [not to enlighten the people, but to keep them unknowing] (Wang, 2011, ch. 65, p. 173, authors’ translation). The accompanying Wang Bi commentary on chapter 65 interprets this language through concerns with cunning and preserving simplicity (Wang, 2011, p. 173). That interpretation complicates a literal reading but does not settle modern questions about access to knowledge and employee voice. These passages mark the distance between an ancient politics of order and contemporary employment relations. The present framework draws a limited insight from wuwei—the leader’s discipline in regulating control—without treating every political claim in the Laozi as a norm for organizations. Its modern normative commitments rest on arguments about autonomy, well-being, and responsibility, not on textual ancestry alone.

## 3 Distinguishing restraint from leadership withdrawal

The relevant distinction is whether leadership responsibilities remain fulfilled when intervention is restrained. Laissez-faire leadership is commonly defined not merely by low activity, but by avoidance, delay, or absence when leadership is needed (Skogstad et al., 2007, 2014). Two leaders may intervene with equal frequency while differing sharply in responsiveness: one has clarified authority, provided resources, and remains available; the other neither decides nor enables others to decide. A conditional account should therefore describe behavior and situational need before evaluating outcomes. This avoids defining wuwei as whatever works and laissez-faire leadership as whatever fails.

Autonomy-supportive and empowering leadership are close comparators. Autonomy support includes taking employees’ perspectives, providing meaningful choices, and reducing controlling pressure; empowering leadership includes power sharing, participation, and support for competence (Gagné and Deci, 2005; Slemp et al., 2018; Lee et al., 2018). These traditions already cover much of the supportive conduct considered here, and empowering behavior can both enable and burden employees (Cheong et al., 2016). Wuwei adds an explicit question about the leader’s continuing authority to intervene: what justifies taking over an authorized decision, and when should control be returned? The proposed extension concerns the timing and justification of intervention, including relinquishing claims to employees’ accomplishments. These concerns are compatible with existing theories; their value lies in specifying a problem for analysis, not establishing a separate leadership style.

Control and support should consequently be described independently of their expected effects. Controlling intervention includes unnecessarily reclaiming delegated decisions, substituting a leader’s preference for an employee’s authorized judgment, or adding approvals unrelated to agreed risks. Justified intervention can instead be assessed against previously stated task boundaries, safety criteria, and escalation signals. A restraint-oriented assessment asks whether intervention is proportionate, whether its rationale is explained, and whether decision authority returns when the triggering condition ends. Support remains observable through resources, response channels, and responsibility arrangements. Table 1 summarizes the conceptual comparisons.

**Table 1:** Conceptual comparison of low-control leadership approaches.

Approach	Core concern	Response when leadership is needed	Relationship to this framework
Supported restraint	Justification, timing, and reversibility of intervention while responsibility continues.	Provides support, intervenes proportionately, and returns authority when the triggering condition ends.	Proposed philosophical lens on intervention episodes; incremental validity remains untested.
Laissez-faire leadership	Avoidance or absence when leadership is needed.	Response is delayed, unavailable, or unclear.	Shares low visible activity but differs in fulfilled responsibility.
Autonomy-supportive leadership	Volition, perspective taking, and reduced controlling pressure.	Provides choice, rationale, and acknowledgment.	Strong overlap; supported restraint makes the justification and ending of intervention an explicit focus within this compatible tradition.
Empowering leadership	Power sharing, participation, and competence support.	Delegates authority and supports capability.	Overlap in discretion and support; supported restraint highlights temporary reintervention and return of authority after delegation, without claiming this concern is exclusive to wuwei.

For example, two managers may delegate equally and provide similar choices, yet only one returns authority after a temporary safety intervention. Examining such episodes could reveal differences obscured by aggregate leadership ratings. This is a testable analytical advantage, not evidence of incremental validity. If established measures explain these patterns fully, wuwei remains a philosophical lens rather than an additional construct. Decision latitude concerns endorsed action within legitimate constraints, not simply the number of choices or the absence of management.

## 4 Four supportive conditions

The four conditions are selected by asking what organizational obligations must be considered when direct control recedes. Role clarity specifies the legitimate scope of action; resources provide the means to act; accessible guidance supplies assistance when difficulties arise; accountability transparency allocates responsibility for decisions and their consequences. Each addresses a different potential failure of delegated discretion: uncertainty, incapacity, unavailable help, or displaced blame. This functional selection draws on research on role ambiguity, workplace resources, autonomy with structure, and accountability (Skogstad et al., 2014; Nielsen et al., 2017; Jang et al., 2010; Hall et al., 2017). It is a provisional, revisable framework rather than an exhaustive taxonomy or a deduction of four dimensions from the Laozi. Support may come from peers, organizational systems, or work design as well as supervisors.

Clear responsibilities make discretion usable by specifying decision rights, coordination interfaces, and escalation routes. Employees need to know which decisions they may take and which obligations remain with leaders. Avoidant leadership’s association with subsequent role ambiguity illustrates the risk of low intervention without this structure (Skogstad et al., 2014). Clarity should define outcomes and constraints without prescribing every step. Evidence that autonomy support and structure can coexist in education provides a conceptual parallel, although transfer to employment requires testing (Jang et al., 2010).

Adequate resources allow employees to act on formally available choices. They include time, information, tools, staffing, expertise, and coordination authority. Delegation without these means may increase workload and personal exposure. Job demands–resources theory and workplace-resource research support examining resources in relation to energy and strain, without validating this four-condition framework (Bakker and Demerouti, 2017; Nielsen et al., 2017). Resource adequacy must be judged relative to demands and unequal access: identical discretion does not confer identical capacity to act.

Accessible guidance enables assistance without routinely taking over decisions. It includes usable question channels, predictable response times, agreed feedback points, and help calibrated to task familiarity. Employees should be able to request assistance without being treated as incompetent. Mentors, colleagues, or technical systems may provide guidance, but leaders remain responsible for ensuring access. Formal availability and perceived safety of help-seeking require separate assessment. Guidance accessibility is prioritized in the initial empirical model because responsiveness most directly distinguishes retained support from withdrawal when visible intervention decreases. Accessibility describes an available resource; withdrawal records nonresponse to a specific need and should not simply be measured by reversing an accessibility score.

Accountability transparency preserves leadership obligations when discretion is distributed. It makes visible who authorizes action, supplies resources, reviews risk, and bears responsibility when circumstances change. Stable, stated evaluation criteria constrain retrospective blame shifting. Accountability can have constructive or adverse consequences, depending on expectations and evaluation practices (Hall et al., 2017). Here transparency concerns the visibility of responsibility arrangements, including leaders’ duties; it does not guarantee organizational justice. Employees must also be able to question decisions without accountability becoming punitive control.

Other influences remain important. Trust and leader–member relationships may shape how support is interpreted; workload affects resource adequacy; competence affects guidance needs; team interdependence affects coordination demands. Organizational justice concerns whether arrangements are fair, beyond whether they are transparent. These factors are not interchangeable with the four conditions. They can be antecedents, contextual influences, or competing explanations, depending on the research question. The four were chosen as modifiable arrangements for making discretion workable, rather than as a ranking of all possible determinants of well-being.

The conceptual framework is broader than the initial test. The first study should vary guidance accessibility while keeping role clarity, resource adequacy, and accountability arrangements adequate and comparable. This restriction isolates a theoretically relevant contrast; it does not establish that the other conditions are universally necessary or unimportant. Subsequent studies can examine them separately. A composite support score and exhaustive interactions among all four dimensions are therefore not proposed for initial testing.

## 5 Psychological mechanisms and testable propositions

Autonomy satisfaction is the principal candidate mechanism. In self-determination theory, autonomy concerns willingness and self-endorsement rather than working alone or escaping constraints (Gagné and Deci, 2005; Van den Broeck et al., 2016; Deci et al., 2017). Lower controlling intervention with accessible help may support this experience and, in turn, work vitality (Slemp et al., 2018). Withdrawal of necessary guidance or coordination remains a distinct predictor: its possible association with emotional exhaustion through role ambiguity belongs to a separate adverse pathway. Reducing coercion and withdrawing support must not be equated.

Psychological safety concerns interpersonal risks of speaking or seeking help (Edmondson, 1999; Frazier et al., 2017); perceived organizational support concerns whether the organization values employees and cares for their well-being (Kurtessis et al., 2017). Both are plausible alternative mechanisms for later studies, not additional mediators in the initial model. Later comparisons should separate individual perceptions from team climate and test when supervisor conduct is attributed to the organization.

Work vitality is the primary outcome for the initial model because it captures positive energetic functioning. Emotional exhaustion is a separate strain outcome for the adverse-pathway extension. Neither is a proxy for performance, and effects need not mirror each other. Studies should prespecify their outcome and time horizon rather than include every possible indicator of well-being.

Proposition 1. Holding role clarity, resource adequacy, accountability arrangements, task demands, and employee experience comparable, the association between lower controlling intervention and autonomy satisfaction is expected to be more positive when guidance is accessible than when it is difficult to obtain.

Proposition 2. Lower controlling intervention is expected to relate indirectly to greater work vitality through autonomy satisfaction, with a more positive indirect association when guidance is accessible. This is the primary conditional process to test; the separate withdrawal–ambiguity–exhaustion pathway is reserved for a subsequent study.

Proposition 3. In subsequent tests, higher task risk or lower employee experience is expected to increase the importance of accessible guidance and clear escalation criteria for autonomy satisfaction under reduced routine intervention. Risk and experience should be examined separately before testing their joint effects. Timely, explained, and proportionate intervention can coexist with autonomy.

An initial factorial scenario experiment could manipulate controlling intervention and guidance accessibility, assess autonomy satisfaction, and prespecify vitality as the outcome. Other support conditions would remain constant. Such an experiment tests immediate responses; mediation and sustained well-being require longitudinal or additional experimental evidence. Later studies could examine the adverse pathway and each boundary condition separately, then compare alternative mechanisms. Event-based employee–supervisor data could assess whether intervention follows stated criteria and whether authority is returned afterward. Comparisons with established autonomy-supportive, empowering, and laissez-faire measures should test discriminant and incremental validity before proposing a new scale. Figure 1 separates the primary model from the adverse-pathway extension; it does not require simultaneous estimation of every proposed relationship.

**Figure 1:**
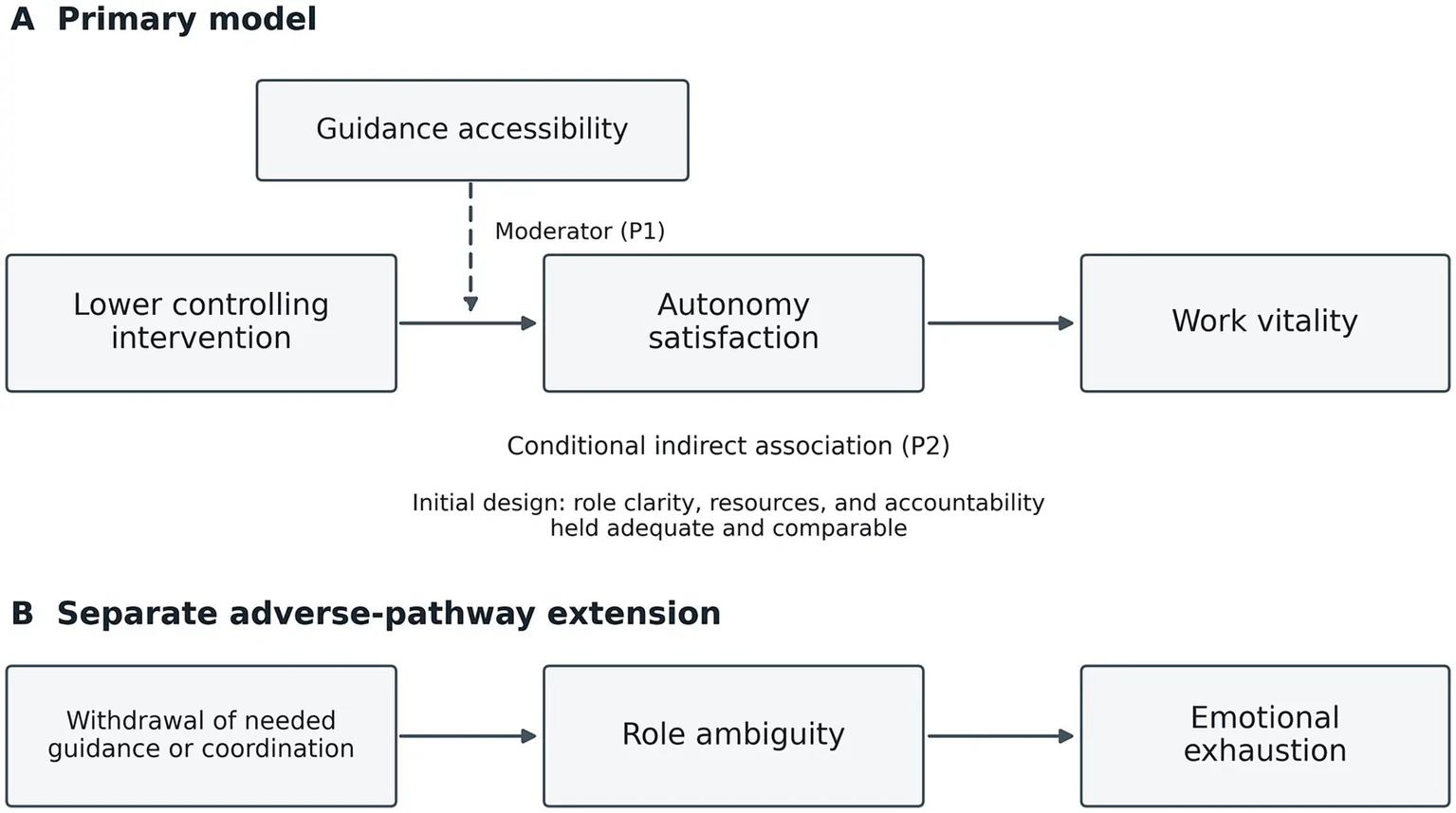
Prioritized hypotheses for staged testing. **(A)** is the primary model: guidance accessibility moderates the association between lower controlling intervention and autonomy satisfaction (P1), yielding a conditional indirect association with work vitality (P2). Role clarity, resources, and accountability arrangements are held adequate and comparable in the initial design. **(B)** is a separate adverse-pathway extension and uses a distinct predictor and outcome. P3, alternative mechanisms, and cultural and institutional comparisons are reserved for subsequent studies. Arrows represent hypothesized associations; no causal relationships are established by this Perspective.

## 6 Discussion

The practical implication is to redesign how leaders remain available while limiting intrusive control. Before delegating, leaders can agree on decision rights, expected outcomes, and escalation conditions. During work, they can check whether resources and response channels remain sufficient without reclaiming routine decisions. Afterward, they can review outcomes using previously stated responsibilities rather than retrospective blame. New employees and high-risk tasks may require more guidance and monitoring, but increased involvement need not eliminate explanation, voice, or reasonable choice. Employee well-being should remain an independent criterion rather than being inferred from performance alone.

Cultural expectations may alter the interpretation of restraint. Where employees expect leaders to authorize action explicitly, reduced instructions without a clear explanation may signal indifference or expose employees to perceived sanction. We therefore expect explicit authorization and safe help-seeking to matter more in such settings. Where independent judgment is established, routine intervention may more readily be interpreted as distrust. These are contextual hypotheses, not claims that national groups are homogeneous. Studies should measure expectations about hierarchy and discretion directly, examine variation within countries, and establish measurement comparability before cross-context comparisons.

Institutional arrangements also determine legitimate intervention. Safety-critical or regulated work may require checks and escalation that cannot simply be delegated away. In interdependent teams, one employee’s discretion affects others, making coordination necessary. Conversely, reliable professional standards and appeal channels may supply support beyond the supervisor. Comparisons should therefore specify oversight obligations, coordination demands, and protection for employee voice, rather than using country or industry labels as explanations.

Related work illustrates this institutional diversity: Zhu (2025) connects wuwei and Buddhist compassion with AI-enabled nonprofit governance. That solo-founder design raises questions about where discretion and responsibility reside; its transfer to employee–supervisor relationships and employee well-being requires separate evidence.

The framework has important limitations. Its selective reading cannot settle the historical meaning of wuwei. Capable employees may elicit lower intervention, while work design may explain both support and well-being. Self-reports risk common-method and attribution bias. The four conditions may have unequal or additive effects, and guidance may not prove the strongest moderator. Existing leadership constructs may fully explain the proposed relationships. These possibilities warrant revision rather than protecting the framework from contrary evidence.

Wuwei directs attention to how leaders govern their intervention while sustaining others’ capacity to act. Its contribution is to connect the justification, timing, and reversibility of intervention with continuing support obligations. A focused initial test and subsequent contextual comparisons can establish whether this perspective clarifies employee experience beyond existing leadership accounts.
